# Cyclic magma recharge pulses detected by high-precision strainmeter data: the case of 2017 inter-eruptive activity at Etna volcano

**DOI:** 10.1038/s41598-019-44066-w

**Published:** 2019-05-17

**Authors:** Gilda Currenti, Alessandro Bonaccorso

**Affiliations:** Istituto Nazionale di Geofisica e Vulcanologia – Osservatorio Etneo, Sezione di Catania, Italy

**Keywords:** Natural hazards, Volcanology

## Abstract

Unprecedented ultra-small strain changes (~10^−8^–10^−9^), preceding and accompanying the 2017 explosive-effusive activity, were revealed by a high precision borehole strainmeter at Etna. No pre- or co-eruptive deformation was detected by the GPS measurements, which often fail to detect ground deformation engendered by short-term small volcanic events due to their limited accuracy (millimetres to few centimetres). Through the analysis and detection of ultra-small strain changes (few tens of nanostrain), revealed by filtering the raw data, a significant time correspondence with the eruptive activity is observed. For the first time, cyclic fast exponential strain changes, preceding the onset of eruptive events, with a timescale of about 2–7 days, were detected. These variations are attributable to the expansion of the shallow magma reservoir, which is replenished with new magma from depth during the inter-eruptive periods. Interpreting the strain changes in terms of pressurization/depressurization of the chamber due to the cyclic influx and withdrawal of magma, allows placing some constraints on the magma recharge volume rate. A Finite Element model has been developed to simulate the temporal evolution of the strain changes generated by the re-pressurization of a spheroidal magma source using a dynamical approach. An average total mass budget of about 1–2 × 10^9^ kg, which is in the range of the erupted mass, is estimated to be accumulated within a shallow vertically elongated magma chamber during the inter-eruptive periods. Such evidence demonstrates that the near-real time analysis of strainmeter records is remarkable for its ability to record small transients and highlight recharging phases preceding eruptive activity, which would go undetected with other current methodologies. Under these conditions, the ability to simulate inter-eruptive periods offers an opportunity to estimate the magma recharge rate with important implications for volcano hazard assessment.

## Introduction

Inflation/deflation cycles in ground deformation are associated with periods of accumulation/withdrawal of magma in storage zones, which expand/contract their volumes under pressure changes due to the transfer of magma batches. Generally, long-term inter-eruptive inflations and short-term co-eruptive deflations, have been observed at different volcanos worldwide by exploiting the results from the analysis of diverse geodetic data such as GPS, DInSAR and levelling^1,2^. However, due to the limited accuracy of these geodetic techniques (millimetres to few centimetres), they often fail to detect ground deformation engendered by small short-term volcanic events. This limitation arises from different possible causes such as: (i) the supplying magma storage is too shallow and, since the deformation pattern is narrow and limited to the summit area, the station geometry might not be dense enough to resolve the associated deformation, (ii) the eruptive event is short-lasting and requires the processing of high rate data with limited signal-to-noise ratio, (iii) the volume change of the magma storage is too small to engender detectable ground deformation. If this is the case, high precision ground-based instruments, such as borehole tiltmeters and strainmeters, may help detect ground deformation and give insights into magma storage volume changes in near-real time for a quantitative estimation of magma accumulation and release^3–9^.

Exemplary case studies of rapid co-eruptive depressurization have been reported at Etna volcano from 2011 to 2016, thanks to more than fifty lava fountain eruptions occurring in this period^10,11^. These lava fountains denoted repeated eruptions with brief duration (from few to several hours), small emitted volumes (~2.5 × 10^6^ m^3^) but with dangerous effects and considerable hazard due to the high explosivity producing a sustained eruptive column and wide ash plumes^12^. No co-eruptive deformation has normally been detected by GPS measurements during the tens of short-term lava fountain episodes occurring in 2011–2016. On the other hand, high precision borehole strain-meters have been capable of detecting co-eruptive strain changes (about 0.2 µstrain), clearly identified in the raw data and associated with short-lasting lava fountaining episodes. The borehole strainmeter provided a tool to infer the source feeding the explosive activity^10,11^. They proved to have very high precision and allowed detecting the effect of small and brief transient lava fountains, whose associated strains are in the range of 10^−6^–10^−7^, difficult to detect with other geodetic techniques. The transient strains, sensed by the network of borehole dilatometers during the lava fountain events, highlighted the contraction of a shallow magma plumbing system, where bubble-rich magma is trapped and then violently released. The periodic nature of these events indicated that the shallow magma storage producing and propelling them is incapable of accumulating large magma volumes and leads to frequent episodes with a fairly constant balance between the refilling and the erupted magma in dynamic equilibrium^10^.

**Figure 1:**
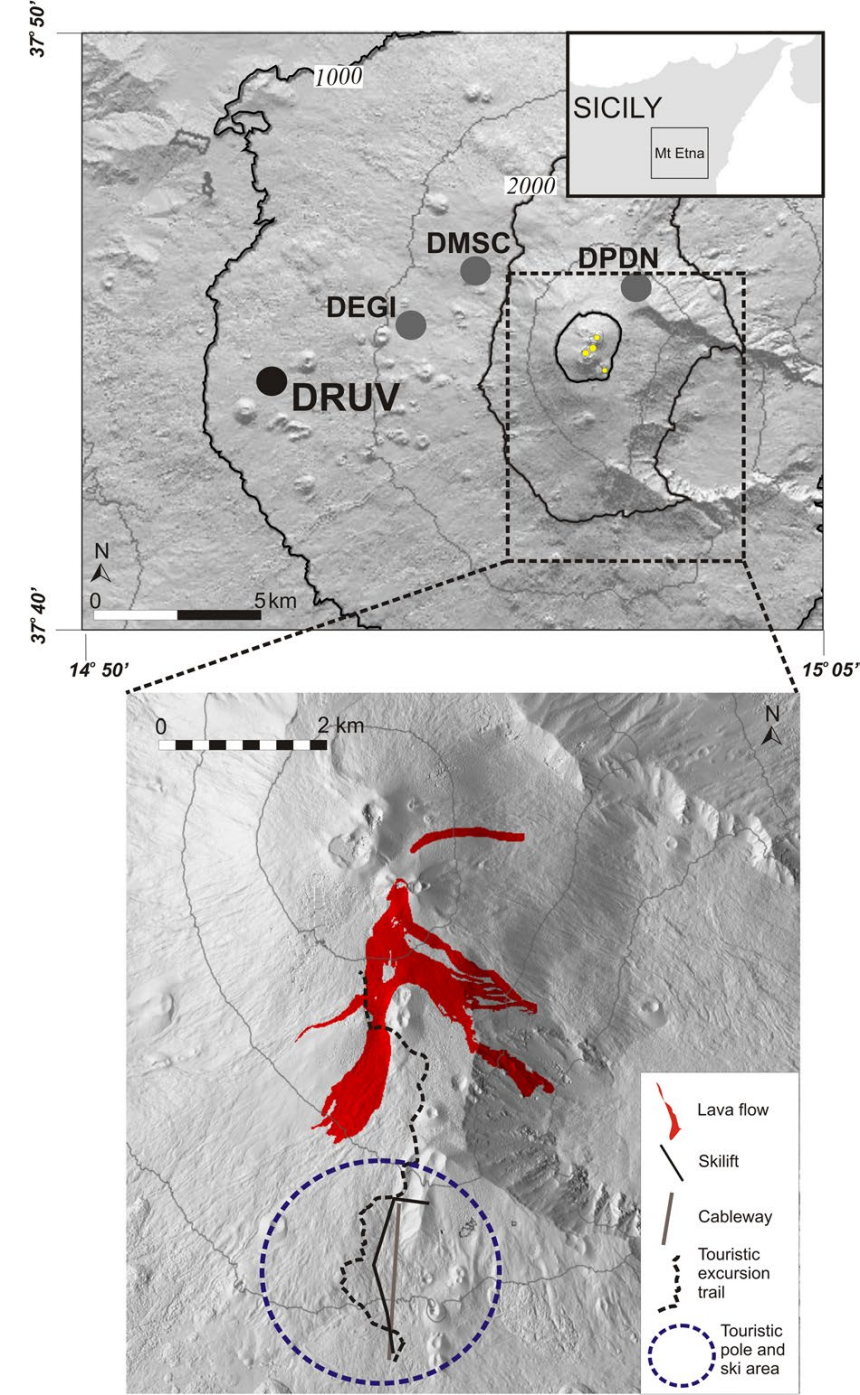
Map of Etna volcano. Strainmeter network consisting of four Sacks-Evertson dilatometers (circles) with the DRUV station used in this study (top). The inset at the top right shows the position of Etna volcano in the eastern part of Sicily (Italy; the rectangle indicates the area enclosed by the top panel). The bottom figure shows the area on the southern flank that is heavily visited by tourist together with the lava flows of March-April 2017^23^.

After the last lava fountains in May 2016, the eruptive activity resumed in February-April 2017 with several distinct eruptions characterized by Strombolian activity and lava flows from the summit crater area (Fig. 1). This time the explosive activity was less powerful than the previous lava fountains of 2011–2016, but the repeated effusive activity generated lava flows directed down the southern flank that is a frequently visited tourist area (Fig. 1), therefore raising serious concern. In particular, two main questions arise: first, can these kinds of modest but dangerous eruptions be forecasted before their onset so as to improve the alert timing? Second, can the accumulation and emission of magma volume be estimated to establish the balance between inflow and outflow, which may provide hints on the duration and intensity of an ongoing eruption? Indeed, so far only co-eruptive volcanic tremor increases and negative strain changes, that correspond to expansion of the rocks engendered by magma chamber de-pressurization, have been observed in time correspondence with the onset of lava fountaining events^10,13^. No significant clear geophysical variations have been reported during the inter-eruptive periods. Therefore, the main challenge to answer to those questions is to discover if possible signatures of recharging preparatory phases could be unmasked from the analysis of available geophysical data.

Here, through the analysis, detection and modelling of small strain changes (few tens of nanostrain), hidden in the raw data, we demonstrate the capacity of a high-precision strainmeter to reveal cyclic inflation and deflation cycles, undetectable in other geodetic signals and preceding and accompanying the eruptive events. For the first time, tiny, but very accurate, nanostrain changes have shown a distinct indication of recharging preparatory phases during inter-eruptive periods. The extraordinary accuracy of the data allows discerning fast recharging phases, which can immediately be used to estimate not only the timing of eruptive events in the near future, but also to evaluate their accumulated volume. This is precious information in terms of both hazard evaluation and mitigation.

### Strain changes accompanying eruptive activity

**Figure 2:**
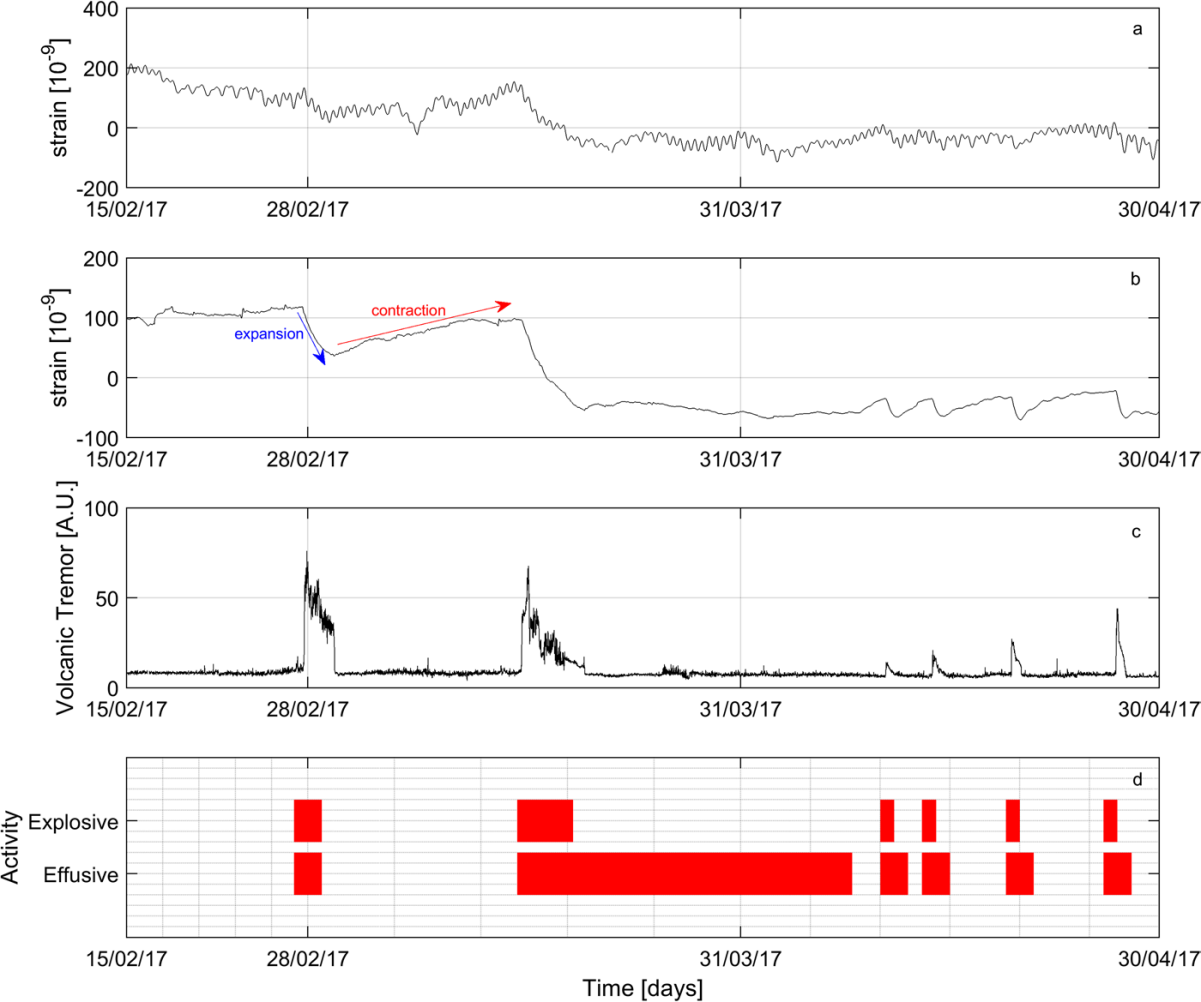
Strainmeter data and eruptive activity at Etna from 15 February to 30 April 2017. (**a**) Raw strain data at DRUV station. A negative strain change corresponds to expansion of the surrounding medium. (**b**) Residual volumetric strain variations after filtering processing (for details, see Fig. S2). (**c**) Co-eruptive events are marked by negative strain changes in correspondence of the increases of the volcanic tremor. (**d**) Small cyclic recharge pulses are clearly recognized in the strain data preceding the onset of the eruptive events. A clear time correspondence is observed with the occurrence of the eruptive activity, reported in the Gantt chart.

We analyse the dataset recorded from October 2016 to April 2017 (Fig. S1) at DRUV station, that, being installed 11 km away from the summit crater at ∼180 m depth in a massive basalt rock (Fig. 1), has very high sensitivity with a signal showing a precision better than 10^−10^ ^14^. As shown by the *in-situ* calibrations^11^, the other strainmeters of the Etnean network (Fig. 1), closer to the summit area, have a lower sensitivity than that at DRUV since they are installed in more compliant rocks with less efficiency in transferring strain from the medium to the sensor. Notwithstanding the high precision of the DRUV strainmeter, no co-eruptive changes are visible in the raw data (Fig. 2a) as occurred for the 2011–2016 lava fountaining events^11^. Because of the weaker volcanic activity, strain signals of volcanic origin that are only a few nanostrain may be obscured by the larger amplitude variations due to tidal strains (expected values of the order of 10^−7^–10^−8^) and atmospheric pressure changes. In order to accurately estimate the contribution of tidal strains and atmospheric pressure changes and, hence, reduce their effect in the strain signal, we used the BAYTAP-G software^15^ (Supplementary Material). The filtered signals (Figs S2, 2b) disclose transient strains, hidden in the raw data, in time correspondence with the resumption of volcanic activity. No variations have been detected in the other strainmeters or at the summit tilt stations (Gambino S., personal communication). Besides rapid transient changes attributable to rainfall (Fig. S2), significant strain variations are associated with the occurrence of six eruptive events, marked by increases in volcanic tremor signal (Fig. 2c). Rapid negative strain variations start and cease simultaneously with the volcanic tremor in the following six time intervals during 2017: 27 February–1 March, 15–19 March, 10–11 April, 13–14 April, 19–20 April and 26–27 April. These intervals correspond to the fast discharge of magma during the eruptive episodes characterized by lava fountains from the summit crater area. The high sensitivity strainmeter measurements have revealed that fairly periodic deflation events accompanying the eruptive episodes are preceded by a quasi-exponential inflation reaching roughly the pre-event strain level. Except for the first event, which was preceded by a remarkably long period of inflation that began in December 2016 (Fig. S2), fast exponential trends are visible before repeated eruptive events. The first explosive event lasted about 3 days. The second event was characterized by an explosive phase of about 4 days. Conversely, the last four consecutive events were briefer, each lasting about two days. Effusive activity not only accompanied but also followed the end of the explosive activity (Fig. 2d). In particular, for the explosive event of 15–19 March, the effusive activity continued for about 30 days after the end of the explosive activity and ended on 8 April. As soon as the effusive activity ceased, a clear positive exponential increase of strain, which denotes contraction of the rock, is observed, which ended at the beginning of the onset of the next explosive activity on 10 April. This cyclic inflation/deflation pattern repeated in the following events. It is worth noting that after the last eruptive event on 26–27 April, no exponential trend has been observed.

After the first long recharge period, which began in December 2016 (Fig. S2), the successive recharge times are much faster. To comprehend the timescale of inter-eruptive inflation events, an estimate of the characteristic times τ_R_ in the strain changes was performed (Fig. S3). We approximated the temporal evolution of the strain variations by $A+B{e}^{-t/{\tau }_{R}}$, which gives a characteristic recharging time ranging between 2–8 days (Table 1). Characteristic discharging times were also fit, providing an estimate, which ranges from a few hours, with a minimum value of about 8 hours obtained for the shorter events, to three days at most for the long-lasting event starting on 15 March (Fig. S3). In addition, a more linear deflation trend accompanies the effusive activity of the second event, which after the end of the explosive activity continued until 8 April.

**Table 1:** Inter-eruptive recharging periods detected by positive strainmeter changes.

Recharge Event	Duration (h)	Characteristic Time (d)	Average Mass (kg)
1 March 2017	311	7.9	2.0 × 10^9^
8 April 2017	44	2.3	1.0 × 10^9^
11 April 2017	59	3.4	1.1 × 10^9^
14 April 2017	114	3.0	1.2 × 10^9^
20 April 2017	163	1.8	1.8 × 10^9^

### Model-based assessment of strain changes

Sawtooth-shaped strain cycles show gradual increases during pressurization and then rapid negative changes accompanying the eruptive events. Rapid co-eruptive deflation is associated with the elastic contraction of the chamber during magma release^10^. Here, we focus on the interpretation and modelling of gradual strain changes during the cyclic inter-eruptive periods. Exponential trends in volumetric strain soon after an eruptive event could be attributable to the re-expansion of the magma chamber due to: (i) influx of new magma from a deeper source and (ii) viscoelastic relaxation of the rocks surrounding the chamber^16^. On the latter point, around a magma chamber, high temperatures make the rocks deviate from their elastic regime and their responses to pressure changes are better represented by viscoelastic rheology, which engenders time-dependent deformation^17,18^. To estimate the amplitude and temporal evolution of the strain changes due to the re-expansion of a magma chamber, a numerical viscoelastic shell model was set up. A Finite Element (FE) model has been developed to compute the strain changes generated by the re-pressurization of a spheroidal magma source in a Maxwell viscoelastic shell^16^ (Fig. S4). We use the numerical approach to simulate the response of the re-expansion of the source, after having supplied magma to the rapid eruptive event. We are aware that the availability of only one measurement point at DRUV is not enough to constrain the model parameters, leading to considerable uncertainty in the estimated volume and mass changes. However, constraints on model parameters could be set on the basis of considerations on magma properties, rock rheology, thermal regime and previous observations. A full description on the definition of the model parameters is reported in the method section. The geometric parameters of the source have also been defined by matching the co-eruptive strain changes with those computed using a FE elastic solution, as already done for similar events attributed to the shallow magma plumbing system at Etna^10^.

The co-eruptive elastic FE model points to a shallow elongated ellipsoidal source at a depth of 0 m b.s.l., in agreement also with the location of the volcanic tremor source^19^. Numerical solutions are necessary to take the topography effect on the strain field into account, which cannot be overlooked due to the shallowness of the source and the relief of Etna volcano.

The amplitude and the timescale of the strain variations during pre-eruption inflation are influenced by model parameters that regulate the viscoelastic relaxation and the mass recharge processes. Evaluating the contribution of these two concurrent processes in the strain changes allows distinguishing between (i) a partial post-eruptive viscoelastic inflation after an eruptive event and (ii) a new recharge phase leading to a future eruption. Distinguishing these processes may help discriminate between an impending eruption or the end of an eruptive episode. Indeed, post-eruptive inflation can occur without recharge of the magma chamber only if the magma is sufficiently incompressible with respect to rock compressibility (i.e. β_m_/β_c_ < 0.8)^16^. In any case, this contribution is not large enough to allow a full recovery of the strain at the pre-eruptive level. To justify the fast inflation, the characteristic time, which is regulated by magma and crust compressibility and the mass recharge rate, should be low. At the source depth (0 m b.s.l.), magma compressibility is expected to be high, because the most significant gas species have already been exsolved (e.g. exsolution pressure of H_2_O ca. 250 MPa; SO_2_ ca. 140 MPa; CO_2_ > 400 MPa). Average value of gas mass fraction at Etna is 3.5 wt%, mainly water and CO_2_. Using theoretical solubility models^20–22^ and average values of phase densities we can derive an average value of magma compressibility. On the basis of these relations, compressibility at shallow depth should be higher than 5 × 10^−10^ Pa^−1^ (Fig. S5). Considering that the average value of rock compressibility β_c_ at shallow depth at Etna is about 6 × 10^−10^ Pa^−1^, the ratio β_m_/β_c_ is much higher than 0.8. Therefore, post-eruptive inflation cannot occur because of viscoelastic relaxation but could be engendered by magma chamber recharge. Indeed, this reasoning supports that the post-eruptive strain changes are generated by time-dependent pressure change due to magma influx.

**Figure 3:**
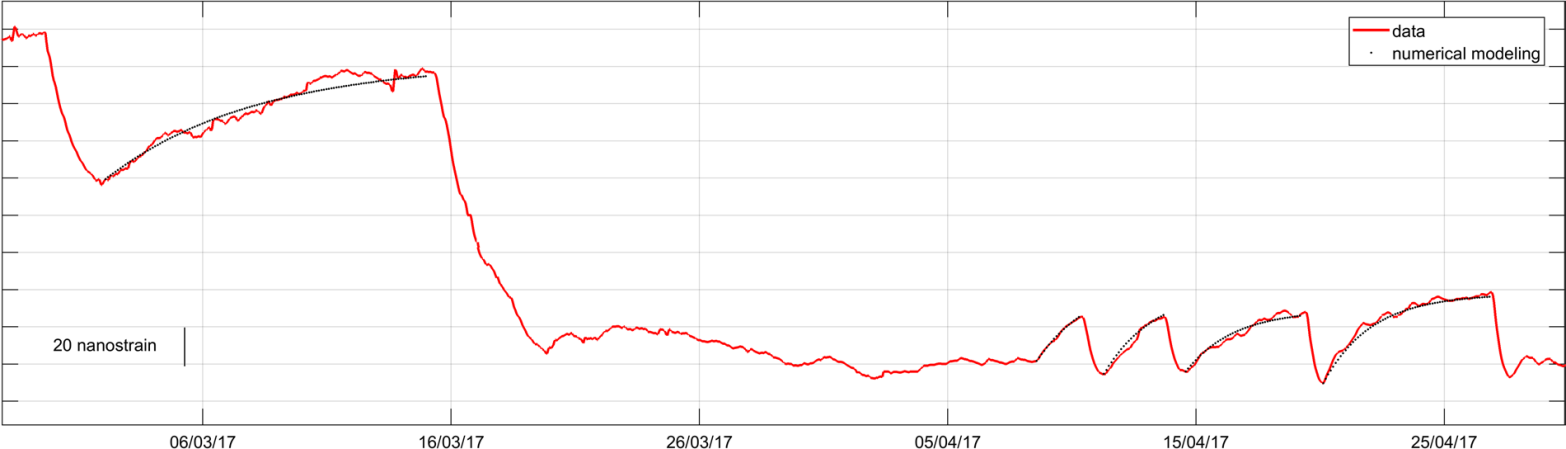
Modelled variation in volumetric strain at DRUV during the detected recharging events. Exponential trends in strainmeter data are explained by modelling mass recharge in the shallow magma plumbing system at Etna. The amplitude and timescale of the strain increases are governed by the overpressure changes and magma flow rate within the chamber.

The model results were compared with strain recorded at DRUV during the recharge pre-eruptive periods (Fig. 3). The good fit is promising to interpret the strain changes in terms of the re-pressurization of the shallow magma chamber. The fitting of the temporal evolution of strainmeter changes at DRUV reveals a mass input rate that evolves exponentially over time for the different events in a range between about 8000 and 500 kg/s, which corresponds to a volume rate of 3-0.2 m^3^/s by assuming an average density of 2400 kg/m^3^ for a bubble bearing magma. Mass inflow into the magma chamber also approximately follows an exponential growth and ceases as the pressure recovers to the pre-eruptive state^16^. The total mass recharge for the different pre-eruptive events varies from 1.0 × 10^9^ to 2.0 × 10^9^ kg (Table 1). During the first two eruptive events, most of the magma accumulated during the long-lasting recharge period since December 2016 (Fig. S2) is released. After the first two eruptive events, the co-eruptive strain negative changes are proportional to the pre-eruptive positive strain increases, indicating an equilibrium between the recharging and discharging phase. Moreover, on average, the recharging magma balances the erupted mass estimated by remote sensing data^23^.

In agreement with modelling results, the data show that the exponential inflation is mainly governed by magmatic recharge. Two main aspects are worthy of note. Firstly, the exponential increase in volumetric strain is observed at the end of the effusive activity on 8 April and precedes the onset of the third event by two days. This is a clear evidence of the inflation of the magma storage due to the accumulation of magma, no longer released by the effusive activity, with a resulting pressurization of the magma chamber. Secondly, at the end of the sixth and last event, no positive strain change is observed and the eruptive activity finally ended.

The onset of a cyclic eruption regime after the first eruptive event implies relatively simple system dynamics with no changes in the effective shallow magmatic plumbing of the volcano, such as the opening of new conduits or vents. The strain changes may be explained by tapping a zoned magma reservoir, where exsolved gases accumulate at the top of the magma source, giving rise to gas-rich fountaining events soon after.

Our results clearly demonstrate that borehole strainmeters are particularly well suited to detect tiny changes even at 11 km away from the summit craters in a safe area, thanks to the high precision and accuracy of the instrument that cannot be matched by other geodetic methods. Combining strainmeter and effusion data with the physical model may allow storage conditions in the magma chamber to be evaluated. In principle, if interpreted in near-real time, the high-precision strain changes may set some constraints on the rate and the intensity of the inter-eruptive inflation and, hence, may assist in estimating eruptable magma volume, eruption onset and duration. This approach can be extremely helpful for hazard mitigation, especially in a situation of frequent eruptive activity in an area visited by numerous tourists.

## Methods

### Data analysis

We analysed seven months of hourly mean strain data recorded from October 2016 to April 2017 (Fig. S1). Preliminarily, the raw data are checked for outliers and instrumental offset that corrupt the signal. The dataset is de-trended to remove a long-lasting drift that affects the measurements during the first years of operation because of mechanical coupling between the medium and the dilatometer. Then, tidal and atmospheric pressure loading effects are filtered from the dataset using the BAYTAPG software package^15^ (Fig. S2).

After filtering the data for tidal and atmospheric pressure components, clear step-like variations are identified in the signal in time correspondence with heavy rain events (Fig. S2). The step-like response to precipitation is ascribed to the rainfall load at the Earth’s surface. Response to rainfall precipitation rises rapidly in the first few hours, and then returns slowly to the pre-rain level over tens of days approximately^24^. A linear regression model is applied to estimate the precipitation response as:

$${y}_{n}=\sum _{j=0}^{k}{c}_{j}{{r}}_{n-j}$$

where *c*_*j*_ are the regression coefficients and *y*_*n*_ and *r*_*n*_ are the strain and rain precipitation time series, respectively. The regression coefficients are estimated on a subset of hourly strain and precipitation data and the optimal model is determined by using the Akaike Information Criterion (AIC). The AIC yields a model order number of k = 300, which indicates a recovery of about 12 days after the rainfall event. Then the regression model is applied to the entire dataset to compute the residual strain changes (Fig. S2).

### Numerical modelling

A FE model is implemented to numerically solve the model equations, which describe the re-pressurization following an eruption from a magma chamber with a viscoelastic shell, on the basis of the formulation described in Segall^16^. Instead of using the analytical solutions^16^, we implemented a numerical FE model to take into account topography and source shape effect. A mass conservation equation is embedded into the viscoelastic system of equations in the FE model by adding a Partial Differential Equation (PDE), which allows computing the time-dependent pressure change δ*p(t)* as a lumped parameter:

$${\rm{\Omega }}[{\rm{\Delta }}{p}_{0}-\delta p(t)]=\rho (V{\beta }_{m}\frac{d\delta p}{dt}+\frac{dV}{dt})$$

where *ρ* is the magma density in the initial state, V is the initial magma chamber volume, *β*_m_ is the magma compressibility and Δ*p*_0_ the jump in the pressure with respect to the pressure state at the time of the co-eruptive event. The mass rate is assumed proportional to the pressure differences through a conductive parameter Ω. The change in pressure is determined by the balance between the magma entering and leaving the chamber and the elasticity of the surrounding rocks. The term on the left side represents the input mass flow rate, which is accommodated in the displaced volume given by the two terms on the right side: the first one, due to the compression of the magma already residing in the chamber, and the second one, generated by the expansion of the chamber wall. Volume changes, required to estimate the temporal evolution of the overpressure, are also numerically solved to take into account the effect of source shape on the effective chamber compressibility^25,26^. The rate of the volume change of the magma chamber is numerically evaluated by integrating the velocity on the chamber wall surface as:

$$\frac{dV}{dt}={\int }_{S}\dot{{\bf{u}}}\cdot {\bf{n}}dS$$

where $\dot{{\boldsymbol{u}}}$ is the derivative of the deformation field, **n** is the unit vector normal to the source wall surface *S*. In the simple case of a spherical source with radius *R*, the volume change rate is approximated by:

$$\frac{dV}{dt}\cong 4\pi {R}^{2}{\dot{u}}_{r}(r=R)$$

where ${\dot{u}}_{r}(r=R)$ is the radial velocity at the source wall, which linearly depends on rock compressibility. Before applying the model to the more general case of a prolate source, a validation with the analytical solution for a spherical source, devised in Segall^16^, has been also performed.

Model parameters are reported in Table S1. Since only one datum is available at the high precision strainmeter of DRUV, no attempts have been made to invert for the many different model parameters, which indeed are set up using findings from volcanological and geophysical constraints on the characteristics of the shallow magma plumbing system at Etna. Particularly, the geometry parameters are those derived from modelling the co-eruptive strain and tilt changes observed during the powerful lava fountaining events in the 2011–2016 period^10,11^. Reasonably, we assume that the same source is active also for the weaker activity analysed here in February-April 2017. The size of the viscoelastic shell is constrained by thermal regime estimates and a temperature dependent Arrhenius-type viscosity law^18^. Assuming a long-lived refilled magma chamber at a temperature of about 1000 °C, the steady-state temperature field distribution is computed by solving a thermal conductive model. At about 200 m from the source wall, the temperature decreases to 700 °C, below which the viscosity, being highly dependent on the temperature state, increases and the response of the medium can be approximated as elastic over the considered timescale^18^.

## Supplementary information

Supplementary Material
