# Supplementary material for: Cyclic magma recharge pulses detected by high-precision strainmeter data: the case of 2017 inter-eruptive activity at Etna volcano
Source: Sci Rep. 2019 May 17;9:7553. doi: 10.1038/s41598-019-44066-w (PMC6525247; doi:10.1038/s41598-019-44066-w)
Supplement: Supplementary file 1 — Supplementary Material [file 41598_2019_44066_MOESM1_ESM.docx]

**Cyclic magma recharge pulses detected by high-precision strainmeter data:**

**the case of 2017 inter-eruptive activity at Etna volcano**

*Gilda Currenti^1^ and Alessandro Bonaccorso^1^*

^1^Istituto Nazionale di Geofisica e Vulcanologia – Osservatorio Etneo, Sezione di Catania (Italy)

Corresponding author: (gilda.currenti@ingv.it)

**Supplementary Figures**


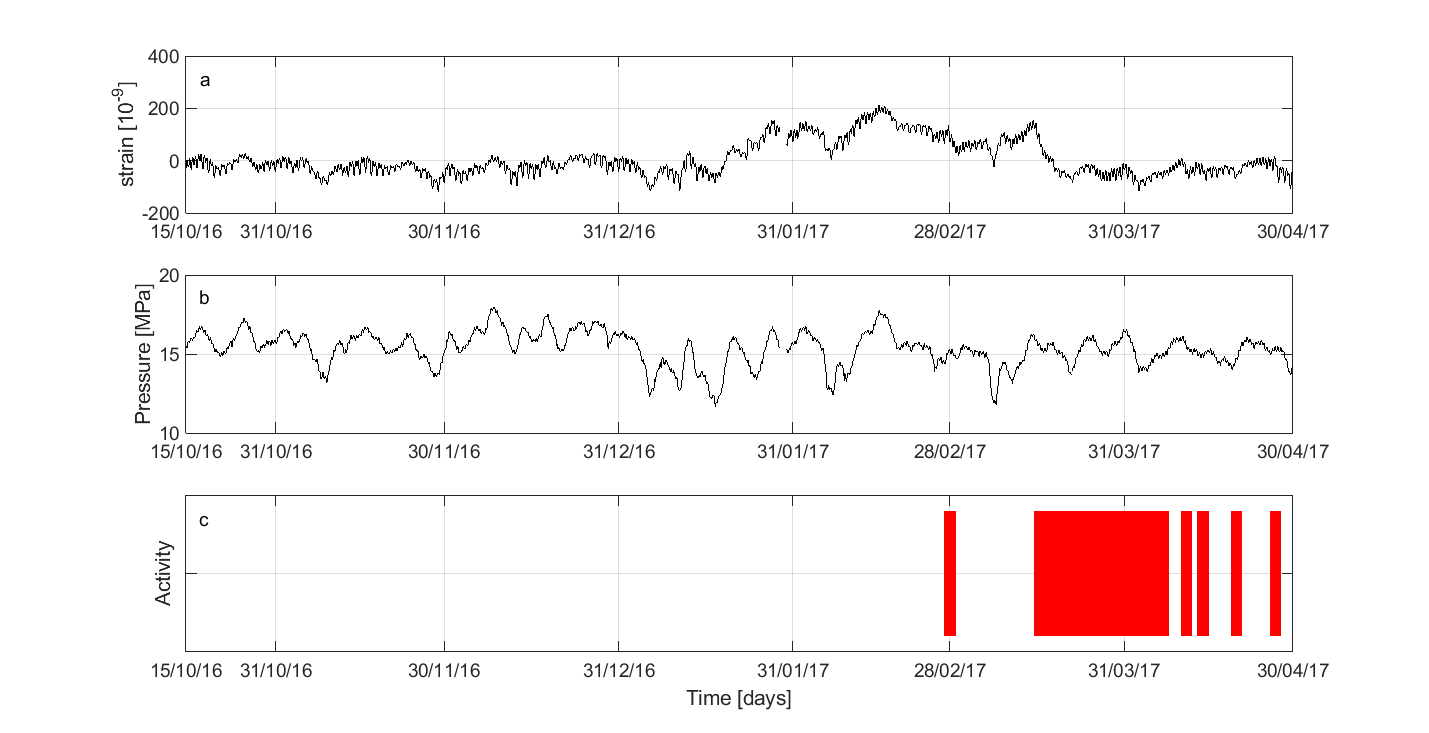


**Figure S1** – Hourly volumetric strain (a) and pressure data (b) at DRUV. Gantt chart (c) of the eruptive activity at Etna from 15 October 2016 to 30 April 2017 (the eruptive periods are in red color).


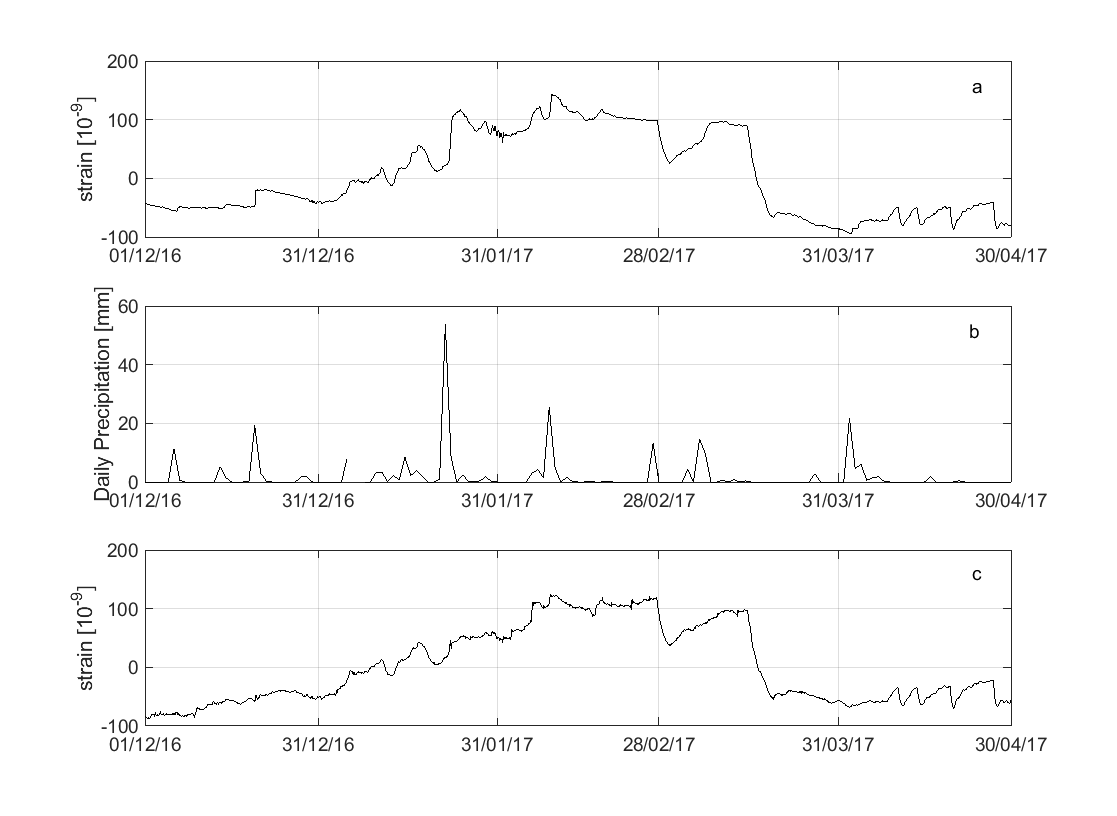


**Figure S2** – Hourly time series of strain (a) after having reduced the contributions of tidal waves and atmospheric pressure by BAYTAPG. Daily rainfall precipitation data (b) acquired from the closest weather station located in Bronte, 8 km away from the DRUV station. Rainfall precipitation data have been provided by SIAS (Servizio Informativo Agrometeorologico Siciliano). Rainfall precipitation and strain data from 15 October to 30 November 2016 are used to find the filter parameter by a minimum least square method. Rainfall effects are quite well reduced (c), even if the weather station is not in the same location of the DRUV strainmeter.


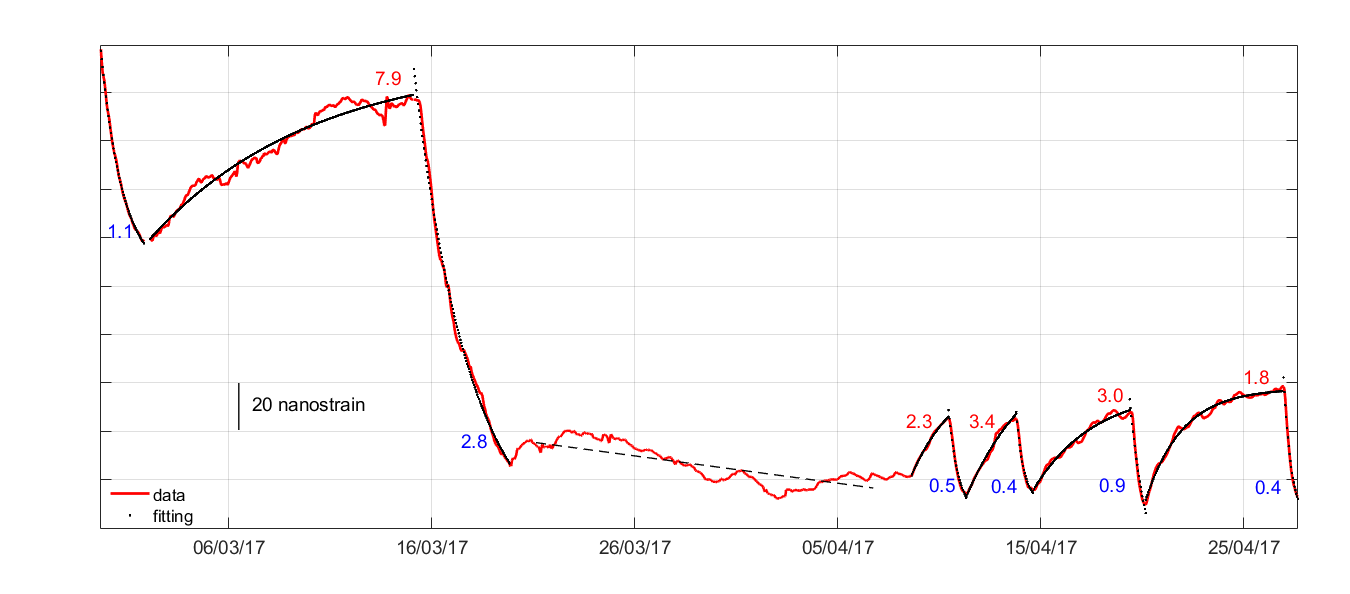


**Figure S3** - Exponential trends preceding and accompanying the eruptive events and their characteristic times (in days) fitted by a first order model during inflation (red values) and deflation (blue value) periods. During the effusive activity (dashed line) strain changes follow a more linear trend.


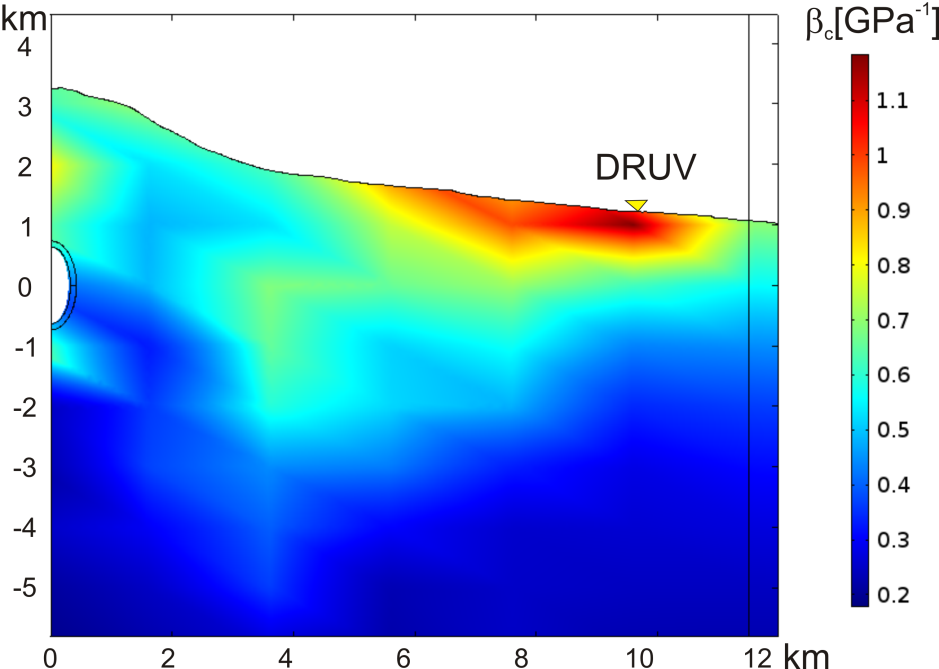


**Figure S4 -** Sketch of the numerical viscoelastic shell model developed under COMSOL (2012). A 3D axi-symmetric model has been set up using a topography profile that runs from summit area toward west in order to describe the average slope of Etna volcano where the DRUV station is located. Full description of the FE viscoelastic shell model can be found in Currenti (2018) and references therein. Average elastic rock compressibility β_c_ at shallow depth is fixed to a value of 6 x 10^-10^ Pa^-1^ by considering elastic rock properties derived from seismic tomography investigations (Del Pezzo et al., 1993; Patanè et al., 2003).

**
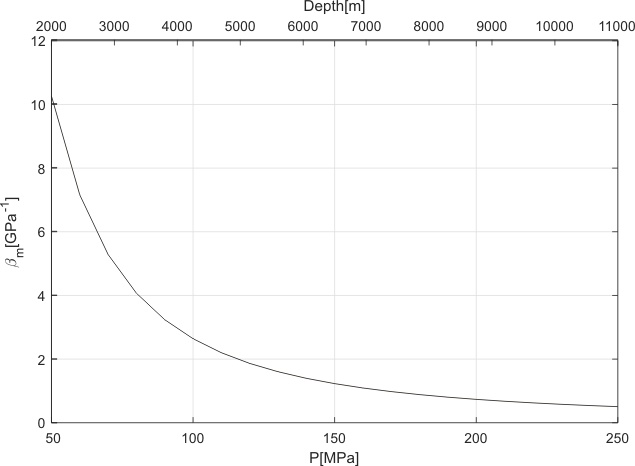
**

**Figure S5 –** Magma compressibility is estimated using the formulation in Johnson et al. (2000) assuming a gas-free melt bulk modulus of 10 GPa, a temperature of 1450 K and a volatile fraction of 0.035 on the average of volatile content at Etna. Similar results are obtained using the model reported in Huppert and Woods (2002), where the exsolved volatile content follows the Henry’s law. Despite the uncertainty on the estimates of β_m_, at the depth of the magma chamber (0 m b.s.l.) the ratio β_m_/β_c_ is much higher than the threshold of 0.8, under whose viscoelastic relaxation may contribute in the inflation of the magma chamber after post-eruptive events (Segall, 2016).

**Supplementary Tables**

**Table S1**. Values of model parameters used to estimate time-dependent strain changes preceding an eruptive event due to re-pressurization of an ellipsoidal magmatic chamber in a viscoelastic shell model.

| **Model Parameters** | **Value** |
| --- | --- |
| Minor ellipse axis (a) | 317 m |
| Major ellipse axis (b) | 634 m |
| Shell thickness (t) | 200 m |
| Viscosity (η) | 10^15^ Pa × s |
| Medium compressibility (β_c_) | 6 × 10^-10^ Pa^-1^ |
| Magma compressibility (β_m_) | 1 × 10^-9^ Pa^-1^ |
| Co-eruptive pressure change (δp_0_) | 1-2 MPa |

**References**

Comsol (2012), Comsol Multiphysics 4.3, 1356 pp., Comsol AB, Stockholm, Sweden.

Del Pezzo, E., De Martino, S., Gresta, S., Martini, M., Milana, G., Patanè, D., Sabbarese, C., 1993. Velocity and spectral characteristics of the volcanic tremor at Etna deduced by a small seismometer array. J. Geophys. Res. 56, 369–378.

Patanè, D., P. De Gori, C. Chiarabba, and A. Bonaccorso (2003), Magma ascent and pressurization of Mt. Etna's volcanic system, Science, 299, 2061 – 2063.
